# Pericoronary Radiomics Signature for Non-Culprit Lesion Progression and Revascularization Decision in NSTE-ACS

**DOI:** 10.3390/diagnostics16091341

**Published:** 2026-04-29

**Authors:** Haidan Zhang, Haichu Wen, Yahui Han, Yifan Wang, Jifang Zhang, Zhen Zhou, Xuelian Gao, Lixin Jia, Lei Xu, Jie Du

**Affiliations:** 1 Beijing Anzhen Hospital, Capital Medical University, Beijing 100029, China; zhanghaidan97@163.com (H.Z.);; 2 School of Pharmacy, Harbin Medical University, Harbin 150081, China; 3 Department of Radiology, Beijing Anzhen Hospital, Capital Medical University, Beijing 100029, China; 4 Beijing Anzhen Nanchong Hospital of Capital Medical University & Nanchong Central Hospital, Nanchong 637003, China; 5 Institute for Biological Therapy, Henan Academy of Innovations in Medical Science, Zhengzhou 450052, China

**Keywords:** non-ST-segment elevation acute coronary syndrome, non-culprit lesion, pericoronary adipose tissue, radiomics, coronary CT angiography

## Abstract

**Background/Objectives**: This study aimed to establish a CCTA-based radiomics model of perivascular adipose tissue (PCAT) to identify high-risk NCLs in patients with NSTE-ACS, potentially facilitating early risk stratification. **Methods**: In this prospective cohort of 542 NSTE-ACS patients, pericoronary adipose tissue (PCAT) radiomic features of non-culprit lesions (NCLs) were extracted from baseline coronary CTA. Patients were assigned to training (*n* = 379) and validation (*n* = 163) cohorts. Machine learning algorithms were applied to develop a radiomics signature (Rad model) to predict 4-year NCL-related major adverse cardiovascular events (MACEs). A combined clinic-radiomics model was constructed to enhance predictive performance. Additionally, the association between the baseline Rad model and longitudinal non-calcified plaque progression (ΔNCPV%/year) was evaluated in a subcohort (*n* = 60) with serial CCTA. **Results**: Over a median 4.0-year follow-up, NCL-related MACE occurred in 84 patients (15.5%). The Rad model (comprising nine features) independently predicted MACE (adjusted hazard ratio, 1.988; 95% CI, 1.753–2.254; *p* < 0.001). In the validation cohort, the combined model yielded higher discrimination for 4-year MACE than the clinical model alone (AUC, 0.793 vs. 0.703; *p* < 0.05). In the serial CCTA subgroup, a higher baseline Rad model was significantly associated with annualized non-calcified plaque volume progression (standardized β, 0.477; *p* < 0.001). **Conclusions**: A CCTA-based PCAT radiomics model is associated with future NCL-related MACE and accelerated plaque progression in patients with NSTE-ACS. This approach may serve as a non-invasive tool for individualized risk stratification.

## 1. Introduction

Non-ST-segment elevation acute coronary syndrome (NSTE-ACS) represents the most prevalent manifestation of acute coronary syndromes globally [1,2]. Although percutaneous coronary intervention (PCI) effectively stabilizes culprit lesions, a formidable long-term risk of major adverse cardiovascular events (MACEs) persists. Landmark natural history studies, such as PROSPECT, have demonstrated that ‘non-culprit’ lesions account for nearly half of recurrent MACEs during long-term follow-up [3,4]. Identifying these biologically unstable NCLs prior to intervention remains a critical clinical challenge [5,6]. Precise risk stratification of NCLs is therefore essential to refine revascularization strategies and tailor secondary prevention for high-risk individuals [7,8].

Current non-invasive and invasive tools for NCL risk stratification have inherent limitations. Invasive coronary angiography (ICA) is confined to luminal stenosis, frequently underestimating the risk of angiographically mild yet biologically active lesions [9,10]. Intracoronary imaging (intravascular ultrasound, near-infrared spectroscopy) can characterize plaque morphology but is invasive, costly, and not suited for screening the entire coronary tree [11]. Fractional flow reserve (FFR), the gold standard for ischemia [12], often fails to identify high-risk NCLs in the NSTE-ACS due to microvascular dysfunction and hemodynamic perturbations; this limitation may explain why FFR-guided revascularization did not consistently improve outcomes in trials such as FULL REVASC [13]. Consequently, a critical gap remains for a non-invasive tool capable of quantifying the inflammatory and biological drivers of plaque progression.

Recent advances highlight the bidirectional crosstalk between the coronary arterial wall and pericoronary adipose tissue (PCAT). PCAT has emerged as a dynamic transducer of vascular inflammation and plaque instability [14,15]. Pro-inflammatory signaling from the vessel wall modulates the composition of adjacent PCAT—a process non-invasively quantifiable via coronary computed tomography angiography (CCTA) [16]. While the perivascular fat attenuation index (FAI) has established the prognostic value of PCAT as a marker of coronary inflammation, it may not fully capture the complex structural alterations within the perivascular space [17,18]. Radiomics-based analysis overcomes this limitation by extracting high-dimensional features that characterize tissue heterogeneity, fibrosis, and microvascular remodeling [19]. Although machine learning-derived radiomics signatures offer incremental value over established imaging markers, their utility in predicting NCL-specific progression remains poorly defined, particularly within the clinically heterogeneous NSTE-ACS population.

Therefore, we sought to develop and validate a CCTA-based PCAT radiomics signature specifically designed to identify “culprit-in-waiting” in patients with NSTE-ACS. Utilizing a prospective cohort with long-term follow-up, we evaluated whether a combined clinic-radiomics model provides incremental predictive value for NCL-related MACE over a baseline clinical model incorporating traditional risk factors. Furthermore, we investigated the association between baseline PCAT radiomics and subsequent plaque progression—quantified by serial CCTA—to provide biological insights into the model’s predictive capacity. Our objective was to establish a robust, non-invasive framework for identifying vulnerable NCLs, providing a biological rationale to guide revascularization decision-making in this high-risk population.

## 2. Materials and Methods

### 2.1. Study Design and Patient Cohorts

This prospective cohort study consecutively enrolled patients presenting with NSTE-ACS [1,2,20], including non-ST-segment elevation myocardial infarction and unstable angina, who underwent successful PCI of the culprit lesion and clinically indicated baseline CCTA between January and December 2021. Patients were randomly assigned in a 7:3 ratio to a model development (training) cohort and an internal validation cohort.

The study protocol was approved by the institutional review board, and written informed consent was obtained from all prospectively enrolled patients. The study was conducted in accordance with the Declaration of Helsinki.

Inclusion criteria were (1) diagnosis of NSTE-ACS; (2) successful PCI of the culprit lesion; (3) availability of analyzable pre-PCI CCTA; and (4) complete clinical follow-up data. Exclusion criteria included: (1) left main coronary artery disease; (2) prior coronary artery revascularization; (3) single-vessel lesion; (4) poor CCTA image quality precluding reliable analysis; and (5) incomplete baseline or follow-up data.

A plaque progression subgroup was defined within the main cohort, consisting of patients who underwent follow-up CCTA for clinical indications at an interval of ≥12 months after the baseline scan for reasons including recurrent atypical chest pain or suspected disease progression. This subgroup was used to evaluate the association between baseline radiomics features and subsequent plaque progression.

### 2.2. CCTA Acquisition, Reconstruction, and Plaque Analysis

All CCTA examinations were performed using a standardized protocol. Image acquisition, contrast administration, electrocardiographic gating, and iterative reconstruction were conducted in accordance with contemporary guideline recommendations, as detailed in the Supplementary Methods.

An NCL was defined as any coronary stenosis lesion with a visually assessed diameter stenosis of at least 50% located in a non-culprit vessel. Quantitative plaque analysis for each NCL was performed using semi-automated software (QAngioCT 3.2.0.13, Medis Medical Imaging 4.0, Leiden, The Netherlands). Percent atheroma volume was calculated as the ratio of total plaque volume to vessel volume multiplied by 100%. Non-calcified plaque volume was defined as plaque voxels with attenuation between 30 and 130 Hounsfield units. The annual changes in non-calcified percent atheroma volume (ΔNCPV%/year) were computed by dividing the change in non-calcified plaque volume percentage by the follow-up interval in years.

### 2.3. PCAT Segmentation and Radiomics Feature Extraction

PCAT segmentation was performed using a custom module in 3D Slicer. For each NCL, the region of interest (ROI) was defined as the adipose tissue extending radially from the outer vessel wall by a distance equal to the diameter of the target vessel, constrained to voxels with attenuation values between −190 and −30 Hounsfield units. Segmentation was independently performed by two trained readers; discrepancies were resolved by consensus.

All images were resampled to isotropic voxels of 0.5 × 0.5 × 0.5 mm^3^ using third-order B-spline interpolation, and voxel intensities were normalized using Z-score transformation. Radiomics feature extraction was performed using PyRadiomics (v3.0.1). A total of 1246 features were extracted from each PCAT region, encompassing first-order statistics, Shape-based (3D), and Texture features (Gray Level Co-occurrence Matrix [GLCM], Gray Level Run Length Matrix [GLRLM], Gray Level Size Zone Matrix [GLSZM], Gray Level Dependence Matrix [GLDM]) from both original and filtered images (Wavelet and Laplacian of Gaussian [LoG] filters).

### 2.4. Feature Stability Assessment and Radiomics Signature Construction

To eliminate redundancy and prevent model overfitting, a rigorous multi-step feature selection strategy was implemented strictly within the training cohort. Inter-reader reproducibility was assessed in 30 randomly selected NCLs using the intraclass correlation coefficient (ICC), and features with an ICC < 0.9 were excluded. Subsequently, features exhibiting strong pairwise correlation (Spearman |r| > 0.90) were eliminated to mitigate multicollinearity. Univariable Cox proportional hazards regression was then applied, identifying candidate features significantly associated with prognostic outcomes (*p* < 0.05).

These candidates were incorporated into a least absolute shrinkage and selection operator (LASSO)-Cox regression model. Ten-fold cross-validation was utilized to determine the optimal penalty parameter, further narrowing the subset. Finally, the remaining non-zero coefficient features were integrated into a random survival forest (RSF) algorithm. The definitive radiomics signature (Rad model) was constructed based on the variable importance (VIMP) scores generated by the RSF. For patients with multiple NCLs, the lesion with the highest angiographic diameter stenosis was selected to represent the patient-level risk.

### 2.5. Endpoint Definitions

The primary clinical endpoint was NCL-related MACE, defined as a composite of cardiovascular death, non-fatal myocardial infarction, or urgent unplanned coronary revascularization attributable to the progression of a previously identified NCL occurring after the index PCI. All events were adjudicated by an independent clinical events committee blinded to radiomics data. In the plaque progression subgroup, the annual changes in non-calcified percent atheroma volume (ΔNCPV%/year) were quantified based on serial CCTA imaging.

### 2.6. Statistical Analysis

Baseline characteristics were compared using standard statistical tests as appropriate. Three Cox proportional hazards models were constructed: a clinical model incorporating baseline clinical risk factors and the Agatston score; a radiomics model incorporating the Rad model score alone; and a combined model integrating the clinical baseline variables with the Rad model.

Model discrimination was evaluated using time-dependent receiver operating characteristic (ROC) curve analysis, with the calculation of the area under the curve (AUC) for the prediction of 4-year NCL-MACE. Differences in AUCs between models were compared utilizing the DeLong test. The incremental prognostic value of the combined model over the clinical baseline was assessed using time-dependent integrated discrimination improvement (IDI) and continuous net reclassification improvement (NRI). Calibration curves were generated, and the Hosmer–Lemeshow goodness-of-fit test was performed to evaluate the agreement between predicted probabilities and observed outcomes.

Kaplan–Meier survival curves were generated stratified by the optimal Rad model cutoff value, determined using maximally selected rank statistics, and differences between groups were compared using the log-rank test. Decision curve analysis (DCA) was performed to quantify the clinical net benefit of each model across a range of threshold probabilities.

In the plaque progression subgroup, multivariable linear regression analyses were conducted to assess the association between the baseline Rad model and imaging-based progression endpoints (ΔNCPV%/year), with adjustment for baseline plaque volume and relevant clinical covariates.

All statistical analyses were performed using R software (version 4.2.2). A two-sided *p*-value < 0.05 was considered statistically significant.

## 3. Results

### 3.1. Study Population and Baseline Characteristics

**Figure 1:**
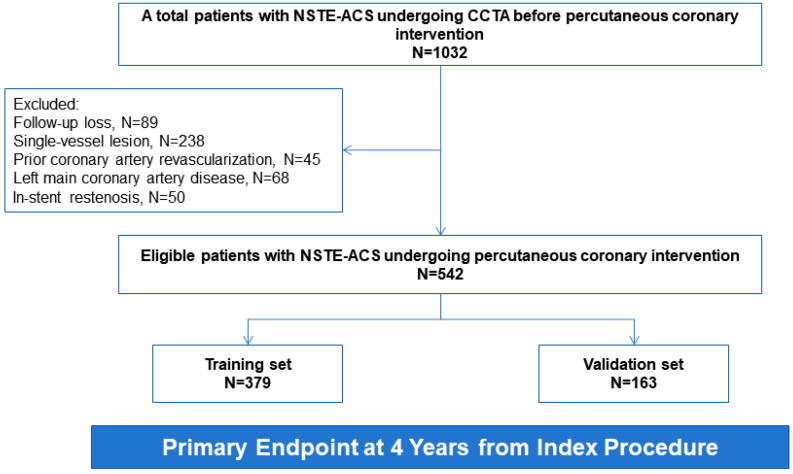
Study Flow Diagram.

A total of 542 patients were included in the final analysis (Figure 1), derived from an initial screening of 1032 patients with NSTE-ACS between January and December 2021. The study population was randomly partitioned into a training cohort (*n* = 379, 70%) and an internal validation cohort (*n* = 163, 30%). The median age of the study population was 60 years (IQR, 54–66 years), and 402 patients (74.2%) were male. Compared with patients without NCL-related MACE, those who experienced the primary endpoint were more likely to have higher BMI levels, and had higher baseline Rad model scores (Table 1). Baseline characteristics between the training and validation cohorts were generally balanced (Table S1).

A subcohort of 60 patients undergoing clinically indicated follow-up CCTA was evaluated for plaque progression (Table S1). The median interval between baseline and follow-up imaging was 2.1 years (IQR, 1.5–2.8 years).

### 3.2. Radiomics Feature Selection and Rad Model Construction

Among the 1246 initially extracted PCAT radiomics features (Table S2), 863 features demonstrated high inter-reader reproducibility (intraclass correlation coefficient [ICC] ≥ 0.9) and were retained for further analysis (Figure S1). Features exhibiting strong pairwise correlation (Spearman |r| > 0.9) were eliminated, refining the pool to 240 independent core features. Univariable Cox proportional hazards regression subsequently identified 11 features significantly associated with prognostic outcomes (*p* < 0.05). These candidates were incorporated into a least absolute shrinkage and selection operator (LASSO)-Cox regression model; utilizing 10-fold cross-validation to determine the optimal penalty parameter, the subset was narrowed to 9 robust predictors (Figure S2A,B). Finally, these 9 features were integrated into a random survival forest (RSF) algorithm. The definitive radiomics signature (Rad model) was constructed based on the variable importance (VIMP) scores generated by the RSF (Figure S2C). All filtering and selection steps were performed strictly within the training cohort.

The final Rad model was constructed using 9 robust features, which were dominated by wavelet-transformed (*n* = 6) and higher-order textures (*n* = 3). Specifically, it comprised features derived from the GLSZM (*n* = 4), GLDM (*n* = 2), and GLCM (*n* = 2), alongside a single Laplacian of Gaussian (LoG)-filtered first-order statistic (Kurtosis).

### 3.3. Predictions of NCL-Related MACE

Over a median follow-up of 4.0 years (IQR: 3.2–4.8 years), 84 NCL-related MACE occurred, yielding an overall incidence of 15.5%. To identify candidate clinical variables for risk adjustment, we performed univariate Cox regression analyses in the training and validation cohorts separately. BMI, eGFR, HDL cholesterol, and Agatston score were consistently associated with NCL-related MACE in the training cohort (all *p* < 0.10; Table 2). These variables, together with age and sex, were included as adjustment factors in subsequent multivariable models. Multivariable Cox analysis confirmed that the Rad model remained an independent predictor of NCL-related MACE after adjusting for these factors (adjusted HR: 1.988; 95% CI: 1.753–2.254; *p* < 0.001; Table 3).

**Figure 2:**
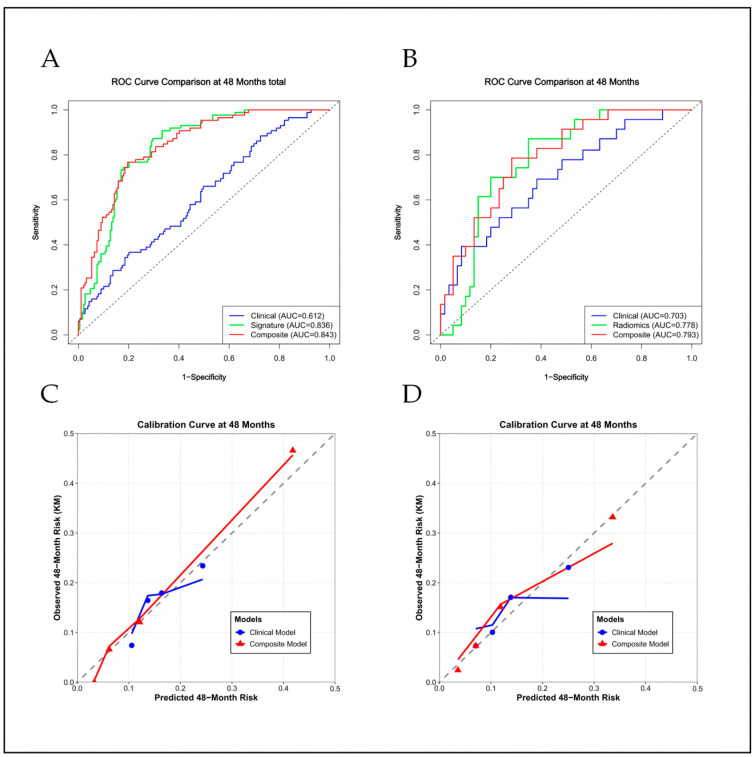
Discriminative performance and calibration of the predictive models for 4-year NCL-related MACE. (**A**,**B**) ROC curves comparing the clinical model, the isolated Rad model, and the combined clinic-radiomics model in the training (**A**) and internal validation (**B**) cohorts. The combined model consistently yields the highest AUC. (**C**,**D**) Calibration curves assessing the agreement between the combined model-predicted probabilities and the actual observed NCL-MACE frequencies in the training (**C**) and validation (**D**) cohorts. The diagonal dashed gray line denotes the ideal reference of perfect calibration. The solid red line represents the bias-corrected performance derived from 1000 bootstrap resamples. The Hosmer–Lemeshow goodness-of-fit test yielded *p* > 0.05 for both cohorts, indicating robust calibration. Abbreviations: AUC, area under the receiver operating characteristic curve.

In the training cohort (*n* = 379), the combined model (integrating clinical variables, Agatston score, and the Rad model) achieved an AUC of 0.843 (95% CI: 0.792–0.892), significantly outperforming the clinical model (AUC: 0.612; 95% CI: 0.533–0.686; ΔAUC: 0.231; 95% CI: 0.162–0.307; DeLong test *p* < 0.001). In the validation cohort, the combined model also showed improved discrimination over the clinical model (AUC: 0.793 [95% CI: 0.684–0.889] vs. 0.703 [95% CI: 0.552–0.819]; ΔAUC = 0.091 [95% CI: 0.025–0.206]; DeLong *p* = 0.016) (Figure 2A,B). The radiomics signature also improved risk reclassification, with more pronounced effects in the training cohort. In the validation cohort, the combined model yielded a continuous NRI of 0.344 (*p* = 0.012) and an IDI of 0.077 (*p* = 0.024) (Table 4).

**Figure 3:**
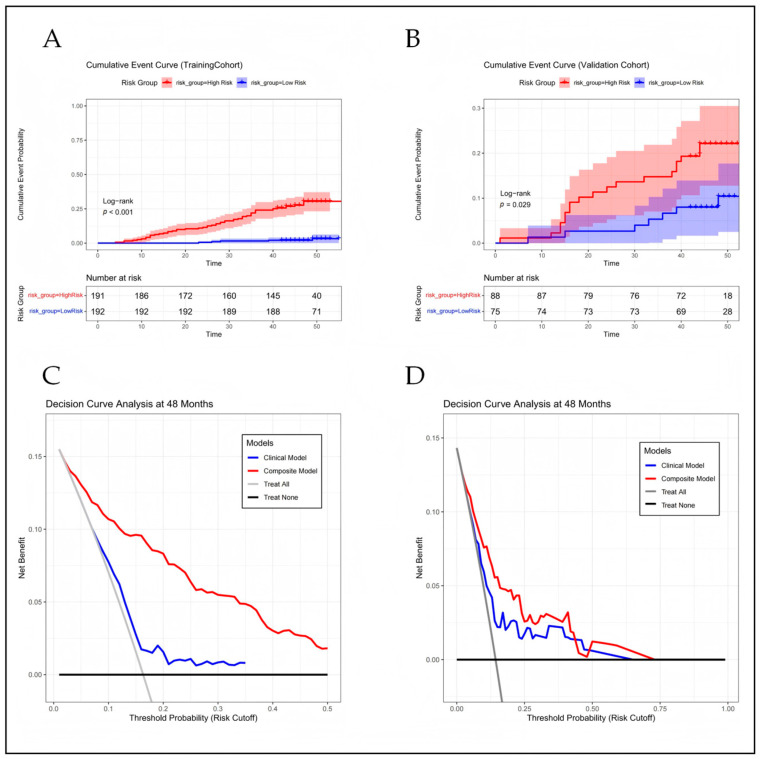
Risk Stratification and Clinical Utility of the PCAT Radiomics Signature (**A**,**B**) Kaplan–Meier curves in training (**A**) and validation (**B**) cohort for cumulative incidence of NCL-MACE stratified by the optimal Rad-score cut-off value, determined using maximally selected rank statistics. Patients with a high Rad model exhibited significantly worse outcomes (log-rank *p* < 0.01). (**C**,**D**) Decision curve analysis (DCA) evaluates the net clinical benefit of the predictive models in the training (**C**) and validation (**D**) cohorts. The horizontal solid black line assumes no patients experience NCL-MACE (“Treat-none”), while the descending solid gray line assumes all patients experience NCL-MACE (“Treat-all”). The combined model (red line) provides a superior net clinical benefit across a broad spectrum of threshold probabilities relative to the clinical baseline model (blue line).

Calibration curves demonstrated robust agreement between the combined model-predicted probabilities and actual 4-year NCL-MACE observations across both cohorts (*p* > 0.05; Figure 2C,D). Kaplan–Meier survival analysis, stratified by the optimal Rad model cut-off, revealed significantly reduced NCL-MACE-free survival in patients with a high radiomics score versus those with a low score (log-rank *p* < 0.001; Figure 3A,B). Decision curve analysis showed that the combined model provided net clinical benefit across a range of threshold probabilities, with curves consistently above those of the clinical model in both cohorts (Figure 3C,D).

### 3.4. Association Between the Rad Model with Plaque Progression

**Figure 4:**
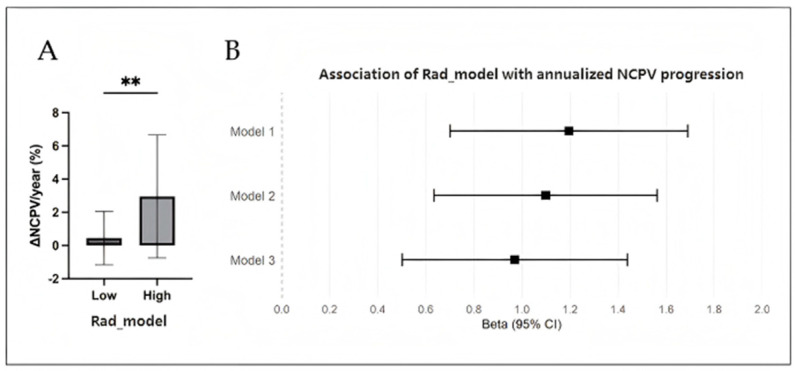
Association Between Baseline PCAT Radiomics Signature and Subsequent Plaque Progression. Analysis performed in the plaque progression subgroup. (**A**) Box plots comparing ΔNCPV/year between patients with high and low Rad model (dichotomized by the median). ** indicates *p* < 0.01. (**B**) Forest plot showing standardized regression coefficients (β) from linear regression models. Model 1: unadjusted; Model 2, adjusted for age and sex; Model 3, Model 2 plus adjusted for baseline NCPV% and clinical risk factors.

Baseline characteristics of the 60 patients in the plaque progression subgroup were comparable to those of the remaining 482 patients in the main cohort (all *p* > 0.05; Table S3), suggesting that the subgroup was representative without significant selection bias. Patients with a high radiomics score demonstrated significantly greater annualized progression of non-calcified plaque (ΔNCPV%/year) compared to those in the low-score group (*p* < 0.001; Figure 4A). Multivariable linear regression established an independent association between the baseline Rad model and ΔNCPV%/year (β = 0.970; 95% CI: 0.501, 1.439; standardized β = 0.477; *p* < 0.001; Figure 4B).

## 4. Discussion

In this prospective cohort of patients with NSTE-ACS, a CCTA-derived lesion-specific PCAT radiomics signature demonstrated robust prognostic value for identifying biologically high-risk NCLs. Our study yields three landmark findings. First, the 9-feature Rad model serves as an independent predictor of 4-year NCL-related MACE, maintaining its prognostic weight after adjusting for anatomical stenosis and clinical risk factors. Second, integrating this radiomics signature with the clinical baseline significantly enhances risk reclassification and discriminative capacity. Finally, the baseline radiomics phenotype correlates strongly with the accelerated longitudinal progression of non-calcified plaque volume (∆NCPV%/year), providing a direct mechanistic imaging correlate for malignant plaque evolution.

### 4.1. Biological Interpretation of the PCAT Radiomics Phenotype: Beyond Mean Attenuation

The biological plausibility of our findings resides in the complex paracrine signaling between the diseased vascular wall and its surrounding perivascular microenvironment [14,17]. Standard attenuation metrics, such as the perivascular FAI, primarily quantify lipolysis and inflammatory edema as a global average of tissue density [18]. However, atherosclerosis is a spatially heterogeneous continuum evolving from acute inflammation to localized fibrosis and microvascular remodeling [21]. By capturing spatial distribution and textural complexity, our Rad model decodes these nuanced architectural alterations that remain imperceptible to conventional FAI. Consequently, the superior predictive power of our radiomics phenotype likely reflects a more integrated assessment of the chronic inflammatory milieu and the resulting structural transformation within the perivascular space [22,23].

Our findings suggest that the dominant radiomics features identified in our model—specifically wavelet-filtered texture features (e.g., GLSZM-Zone Variance, GLDM-Large Dependence Low Gray Level Emphasis), quantify spatial heterogeneity and voxel intensity irregularities. These high-dimensional features likely encode a chronic “fibrosis-remodeling” phenotype characterized by collagen deposition, interstitial irregularity, and pathological neovascularization within the PCAT [24,25]. This paradigm explains the dual predictive capacity of the Rad model: it decodes the cumulative structural “scarring” that standard attenuation averages out, effectively predicting both subclinical non-calcified plaque expansion and subsequent clinical MACE. By integrating the inflammatory “snapshot” with structural remodeling, the signature offers a comprehensive assessment of biological instability.

### 4.2. Comparison with Existing PCAT and Radiomics Research

Considerable progress has been made in establishing PCAT as a non-invasive biomarker of cardiovascular risk, and the present study extends this work in several important respects.

First: From Global Correlation to Lesion-Specific Prognosis

Early investigations, such as the landmark CRISP-CT study, established PCAT FAI as a global marker of coronary inflammation [18]. Our work extends this foundation by shifting the focus toward a lesion-specific prognostic paradigm. Previous research primarily correlated perilesional FAI with surrogate markers of vulnerability, such as OCT-defined thin-cap fibroatheroma [26,27]. In contrast, we targeted a high-risk NSTE-ACS population and utilized hard NCL-related clinical endpoints. This transition from cross-sectional correlation to prospective risk prediction in a volatile cohort significantly elevates the clinical relevance of PCAT assessment.

Second: Distinguishing PCAT Radiomics from Plaque Analysis

The present findings also complement and expand upon emerging radiomics research. Whereas Kolossváry et al. [28] demonstrated the utility of plaque-based radiomics and Lin et al. [29] focused on identifying already ruptured culprit lesions, our model prospectively risk-stratifies angiographically stable NCLs by targeting the perivascular microenvironment. Pan et al. recently validated PCAT radiomics for predicting plaque progression using different machine learning architectures [30]. Building on this, our study extends predictive modeling toward clinical utility in NSTE-ACS. Moreover, Kim et al. showed that PCAT radiomic features correlate with OCT-derived thin-cap fibroatheroma and macrophage infiltration, indirectly supporting the biological plausibility of our findings [31]. While their study used invasive OCT, our serial CCTA observation of accelerated non-calcified plaque progression parallels their microscopic findings. Consequently, by incorporating both clinical events and serial CCTA-defined progression, our work offers a more longitudinal perspective than earlier PCAT radiomics studies.

Third: Independent Value Beyond Angiographic Severity and Global Plaque Burden

Crucially, our study demonstrates that the Rad model addresses the limitations of conventional anatomical assessment. While ICA is the gold standard for defining stenosis severity, its ability to predict which NCLs will lead to future events is limited, particularly for intermediate lesions. Notably, the Rad model remained a robust and independent predictor of NCL-MACE even after adjusting for clinical risk factors and the Agatston score (Table 2). This suggests that PCAT radiomics captures a distinct biological dimension—the localized inflammatory activity—that is not reflected by the degree of luminal narrowing. The significant improvement in model discrimination (AUC increase from 0.703 to 0.793) underscores the importance of integrating biological signatures with anatomical data. Whether this biological information translates into improved clinical outcomes when used to guide revascularization decisions remains to be tested. At this hypothesis-generating stage, the Rad model identifies a candidate imaging biomarker that warrants further evaluation in prospective interventional studies.

### 4.3. Limitations

First, despite internal validation within a prospective cohort, our findings are derived from a single-center experience. External validation in diverse, multicenter populations is essential to confirm the generalizability and cross-vendor reproducibility of the PCAT radiomics signature. Second, radiomics features are inherently sensitive to CT acquisition parameters and reconstruction algorithms. Although we implemented intensity normalization and stringent stability filtering (ICC > 0.9) to mitigate these effects, the standardization of PCAT radiomics across different scanner platforms remains an ongoing challenge for clinical translation [32]. Third, serial CCTA follow-up was clinically driven rather than protocolized. This introduces inherent selection bias, as patients requiring repeat scans frequently present higher pre-test probabilities of disease progression. Despite demonstrated baseline cohort homogeneity (Supplementary Table S3), this symptom-driven methodology cannot be completely mitigated and may overestimate average plaque progression rates. Validation through prospective, protocol-mandated imaging remains essential. Fourth, we did not have intracoronary imaging data (OCT or IVUS) to directly correlate our PCAT radiomics features with established histological markers of plaque vulnerability, such as thin-cap fibroatheroma or macrophage infiltration. Future studies integrating CCTA-based radiomics with invasive imaging are needed to validate the underlying histopathology.

## 5. Conclusions

In this prospective cohort of patients with NSTE-ACS, a CCTA-derived PCAT radiomics signature was independently associated with longitudinal non-calcified plaque progression and MACE. The signature quantifies textural heterogeneity within the pericoronary microenvironment, capturing features of biological plaque instability not reflected by conventional anatomical assessment. PCAT radiomics may serve as a non-invasive biomarker to assist in lesion-level risk stratification.

## Figures and Tables

**Table 1:** Baseline Characteristics Between Patients with and without NCL-related MACE.

	No MACEs (*n* = 458)	MACEs (*n* = 84)	*p*
Age (years)	60.00 (54.00, 66.00)	61.00 (54.75, 67.00)	0.729
Sex (male, %)	336 (73.4)	66 (78.6)	0.386
Hypertension (%)	290 (63.3)	63 (75.0)	0.052
Hyperlipemia (%)	369 (80.6)	68 (81.0)	1.000
Diabetes (%)	163 (35.6)	33 (39.3)	0.600
DBP (mmHg)	78.00 (70.00, 85.00)	77.50 (70.00, 85.00)	0.710
SBP (mmHg)	130.00 (121.00, 141.00)	129.00 (117.00, 138.00)	0.094
BMI (kg/m^2^)	25.62 (23.74, 27.69)	26.69 (24.39, 28.81)	0.014
ALT (U/L)	18.00 (13.00, 25.00)	18.00 (14.75, 28.00)	0.254
AST (U/L)	18.00 (15.00, 22.00)	20.00 (17.00, 24.00)	0.051
Creatinine (μmol/L)	73.50 (64.20, 83.47)	75.30 (64.03, 83.08)	0.954
eGFR (mL/min/1.73 m^2^)	93.28 (83.16, 101.26)	93.94 (87.32, 102.40)	0.284
Glu (mmol/L)	5.99 (5.12, 7.65)	5.99 (5.20, 8.54)	0.530
TG (mg/dL)	1.45 (1.08, 2.06)	1.55 (1.10, 2.20)	0.387
TC (mg/dL)	4.23 (3.42, 5.03)	4.14 (3.53, 5.33)	0.677
HDL (mg/dL)	1.04 (0.89, 1.22)	1.08 (0.91, 1.18)	0.680
LDL-C (mg/dL)	2.31 (1.76, 3.10)	2.18 (1.81, 3.00)	0.905
hs-CRP (mg/L)	1.23 (0.60, 2.82)	1.29 (0.72, 2.91)	0.469
Hemoglobin (g/dL)	6.00 (5.60, 6.80)	6.10 (5.77, 6.80)	0.345
Agatston	346.38 (75.29, 890.26)	377.15 (67.35, 1012.87)	0.872
Rad model	2.55 (1.84, 3.42)	4.58 (3.73, 5.32)	<0.001

Comparison of demographic, clinical, laboratory, and baseline Agatston score between the MACE and NoMACE group. Data are presented as median (interquartile range), or number (percentage), as appropriate. *p*-values were derived using Mann–Whitney U tests, or Chi-square tests. Abbreviations: SBP, Systolic Blood Pressure; DBP, Diastolic Blood Pressure; BMI, body mass index; AST, Aspartate Aminotransferase; ALT, Alanine Aminotransferase; eGFR, Estimated Glomerular Filtration Rate; Glu, Glucose; TG, Triglyceride; TC, Total Cholesterol; HDL, high-density lipoprotein; LDL-C, low-density lipoprotein cholesterol; hs-CRP, high-sensitivity C-reactive protein.

**Table 2:** Univariable Cox Proportional Hazards Analysis for the Prediction of NCL-related MACE in the Training and Validation Cohorts.

Variable	HR in Training Cohort	95% CI	*p* Value	HR in Validation Cohort	95% CI	*p* Value
Age (years)	1.01	0.98–1.04	0.517	0.98	0.94–1.02	0.416
Sex (male)	1.66	0.86–3.19	0.128	0.80	0.33–1.95	0.626
Hypertension	1.52	0.86–2.70	0.147	2.01	0.74–5.40	0.168
Hyperlipidemia	1.16	0.59–2.28	0.673	0.78	0.31–1.98	0.601
Diabetes	1.11	0.66–1.85	0.705	1.29	0.56–2.94	0.548
DBP	1.00	0.98–1.02	0.840	0.99	0.95–1.03	0.730
SBP	0.99	0.98–1.01	0.301	0.98	0.95–1.01	0.146
BMI (kg/m^2^)	1.07	0.99–1.14	0.077	1.15	1.02–1.30	0.024
ALT (U/L)	1.01	0.99–1.02	0.416	1.00	0.97–1.03	0.818
AST (U/L)	1.01	1.00–1.01	0.109	1.01	0.96–1.07	0.578
Creatinine (μmol/L)	1.00	0.99–1.02	0.593	0.99	0.96–1.01	0.369
eGFR (mL/min/1.73 m^2^)	1.00	1.00–1.00	0.009	1.01	0.99–1.04	0.304
Glu (mmol/L)	1.01	0.93–1.09	0.819	1.14	1.02–1.29	0.027
TG (mg/dL)	1.08	0.96–1.22	0.185	1.13	1.02–1.26	0.020
TC (mg/dL)	1.06	0.87–1.29	0.567	1.01	0.75–1.38	0.930
HDL (mg/dL)	1.48	1.05–2.08	0.026	0.47	0.09–2.60	0.387
LDL-C (mg/dL)	0.99	0.93–1.05	0.744	0.83	0.53–1.29	0.411
hsCRP (mg/L)	0.97	0.90–1.05	0.448	1.01	0.92–1.11	0.792
Hemoglobin (g/dL)	1.00	0.98–1.02	0.737	1.01	0.99–1.04	0.340
Agatston	1.00	1.00–1.00	0.045	1.00	1.00–1.00	0.435
Rad model	2.08	1.79–2.40	<0.001	1.57	1.25–1.97	<0.001

Data are presented as unadjusted hazard ratios (HRs) with 95% confidence intervals (CIs). This table summarizes the association between each individual baseline variable and the risk of NCL-related MACE during follow-up.

**Table 3:** Multivariable Cox Regression Analysis for NCL-MACE in the Validation Set.

Model	HR	95%CI	*p*-Value
Model 1	1.885	1.677–2.119	<0.001
Model 2	1.893	1.683–2.128	<0.001
Model 3	1.988	1.753–2.254	<0.001

Results of multivariable Cox proportional hazards models assessing the independent prognostic value of the radiomics signature (Rad model) for NCL-MACE. The Rad model was analyzed as a continuous variable per standard deviation increase. Model 1 included the Rad_model only (unadjusted). Model 2 was additionally adjusted for age and sex. Model 3 represents the final clinical model (age, sex, BMI, eGFR, HDL, Agatston) plus the Rad model. Hazard ratios (HRs), 95% confidence intervals (CIs), and *p*-values are shown.

**Table 4:** Incremental prognostic value of the combined model compared to the clinical model for predicting 4-year NCL-related MACE.

Cohort	Time-Dependent IDI (95%CI)	*p*-Value	Continuous NRI (95%CI)	*p*-Value
Training	0.206 (0.139–0.282)	<0.001	0.506 (0.359–0.615)	<0.001
Validation	0.077 (0.008–0.192)	0.024	0.344 (0.076–0.578)	0.012

The combined model integrates baseline clinical risk factors, the Agatston score, and the PCAT radiomics signature (Rad model). The clinical baseline model incorporates clinical risk factors and the Agatston score alone. Time-dependent integrated discrimination improvement (IDI) and continuous net reclassification improvement (NRI) were computed at the 4-year follow-up endpoint to quantify the incremental risk reclassification capacity.

## Data Availability

Due to ethical and privacy restrictions, these data are not publicly available but can be provided upon reasonable request from the corresponding author, in compliance with institutional policies.

## Supplementary Materials

The following supporting information can be downloaded at: https://www.mdpi.com/article/10.3390/diagnostics16091341/s1, Method S1: Detailed CCTA protocols; Table S1: Baseline Characteristics of the Training and Validation Cohorts; Table S2: Complete List of Extracted Radiomics Features and Their Reproducibility; Table S3: Baseline Characteristics of the Plaque Progression Subgroup; Figure S1: Feature Stability analysis; Figure S2: Feature Selection Process.

## Abbreviations

The following abbreviations are used in this manuscript:

AUC	Area Under the Curve
CCTA	Coronary Computed Tomography Angiography
CI	Confidence Interval
DCA	Decision Curve Analysis
FAI	Fat Attenuation Index
FFR	Fractional Flow Reserve
ICA	Invasive Coronary Angiography
ICC	Intraclass Correlation Coefficient
IDI	Integrated Discrimination Improvement
NCL	Non-culprit Lesion
NCPV%	Percentages of Non-calcified Plaque Volumes
NRI	Net Reclassification Improvement
NSTE-ACS	Non-ST-Segment Elevation Acute Coronary Syndrome
MACE	Major Adverse Cardiovascular Events
PAV	Percent Atheroma Volume
PCAT	Pericoronary Adipose Tissue
ROC	Receiver Operating Characteristic
RSF	Random Survival Forest
