# Supplementary material for: Pericoronary Radiomics Signature for Non-Culprit Lesion Progression and Revascularization Decision in NSTE-ACS
Source: Diagnostics (Basel). 2026 Apr 29;16(9):1341. doi: 10.3390/diagnostics16091341 (PMC13163618; doi:10.3390/diagnostics16091341)
Supplement: Supplementary file 1 [file diagnostics-16-01341-s001.zip › diagnostics-4252615-supplementary.pdf]

**Supplementary Materials**

## Table of Contents

1. **Supplementary Method S1**
2. **Supplementary Tables S1-S3**
3. **Supplementary Figures S1-S2**

## Supplement Method S1

Detailed CCTA protocols:

CCTA examinations were performed using four different scanners: two dual-source CT scanners (SOMATOM Force and SOMATOM Definition Flash, Siemens Healthineers, Forchheim, Germany), a 256-section wide-detector CT scanner (Revolution CT, GE Healthcare, Milwaukee, WI, USA), or a 320-detector row CT scanner (Aquilion One, Canon Corporation, Japan). The scan range covered from 1.0 cm below the tracheal carina to 1.5 cm below the heart. Contrast administration involved an automated real-time bolus-tracking technique to trigger acquisition, targeting the ascending aorta. During the procedure, 0.8 mL/kg of Iohexol 350 (GE Ltd., US) was injected at a rate of 4.0–5.0 mL/s, followed by a 30 mL saline flush to ensure optimal imaging. For all scans, prospective echocardiographic gating was adjusted based on heart rate (HR): 25–55% of the R-R interval for HR > 80 bpm, 30–80% for HR< 80 bpm, 65–85% for HR< 65 bpm. Details of the scan parameters are as follows:

(1) Scanner 1 (SOMATOM Force, Siemens): rotation time: 0.25 s, pixel matrix: 512 × 512, collimation: 2 × 96 × 0.6 mm, and tube voltage: 100 or 120 kV, with automatically selected tube current. The section thickness was 0.6 mm, with a reconstruction increment of 0.6 mm. All raw data were reconstructed using an IR algorithm (sonogram-affirmed iterative reconstruction; SAFIRE; Siemens Healthineers)

with a weight of 3.

(2) Scanner 2 (SOMATOM Definition Flash, Siemens): rotation time: 0.28 s, pixel matrix: 512 × 512, collimation: 2 × 64 × 0.6 mm, and tube voltage: 100 or 120 kV, with automatically selected tube current. The section thickness was 0.6 mm, with a reconstruction increment of 0.6 mm. All raw data were reconstructed using an IR algorithm (sonogram-affirmed iterative reconstruction; SAFIRE; Siemens Healthineers) with a weight of 3.

(3) Scanner 3 (Revolution CT, GE): rotation time: 0.28 s, pixel matrix: 512 × 512, collimation: 256 × 0.625 mm, and tube voltage: 100 or 120 kV, and the Smart mA was applied. The section thickness was 0.625 mm, with a reconstruction increment of 0.625 mm. All raw data were reconstructed using an IR algorithm (adaptive statistical iterative reconstruction-V, ASiR-V™; GE Healthcare) with a weight of 60%.

(4) Scanner 4 (Aquilion One, Canon): rotation time: 0.35 s, pixel matrix: 512 × 512, collimation: 320 × 0.5 mm, tube voltage: 100 or 120 kV, and tube current: 200–400 mA. The slice thickness was 0.5 mm, with a reconstruction increment of 0.5 mm. All raw data were reconstructed using a medium soft-tissue convolution reconstruction kernel (I26f/Bv40).

**Supplement Tables**

Table S1. Baseline Characteristics of the Training and Validation Cohorts

| | Training cohort (n=379) | Validation cohort (n=163) | *p*-value |
|---|---|---|---|
| age (years) | 60.00 (54.00, 66.00) | 61.00 (53.50, 66.00) | 0.669 |
| gender (male，%) | 281 (74.1) | 121 (74.2) | 1.000 |
| Hypertension (%) | 247 (65.2) | 106 (65.0) | 1.000 |
| Hyperlipidemia (%) | 310 (81.8) | 127 (77.9) | 0.353 |
| Diabetes (%) | 135 (35.6) | 61 (37.4) | 0.762 |
| DBP (mmHg) | 77.00 (69.00, 85.00) | 78.00 (71.00, 85.00) | 0.424 |
| SBP (mmHg) | 129.00 (120.00, 140.00) | 131.00 (121.50, 141.00) | 0.261 |
| BMI (kg/m$^2$) | 25.81 (23.88, 27.90) | 25.69 (23.88, 27.89) | 0.731 |
| ALT (U/L) | 18.00 (13.00, 27.00) | 19.00 (14.00, 25.50) | 0.495 |
| AST (U/L) | 19.00 (15.50, 23.00) | 18.00 (16.00, 21.00) | 0.417 |
| Creatinine (μmol/L) | 74.50 (65.50, 83.20) | 71.90 (62.35, 83.90) | 0.248 |
| eGFR (mL/min/1.73m$^2$) | 93.15 (83.48, 100.80) | 95.20 (85.94, 102.43) | 0.131 |
| Glu (mmol/L) | 6.02 (5.16, 7.69) | 5.93 (5.00, 7.75) | 0.376 |
| TG (mg/dL) | 1.52 (1.08, 2.13) | 1.41 (1.08, 2.00) | 0.256 |
| TC (mg/dL) | 4.31 (3.56, 5.14) | 4.09 (3.32, 4.94) | 0.020 |
| HDL (mg/dL) | 1.04 (0.91, 1.23) | 1.02 (0.89, 1.17) | 0.146 |
| LDL-C (mg/dL) | 2.33 (1.87, 3.14) | 2.13 (1.62, 2.96) | 0.028 |
| hsCRP (mg/L) | 1.24 (0.66, 2.87) | 1.22 (0.56, 2.71) | 0.479 |
| Hemoglobin (g/dL) | 6.00 (5.60, 6.75) | 6.10 (5.65, 7.00) | 0.494 |
| Agatston | 343.88 (69.13, 903.00) | 371.62 (83.00, 888.79) | 0.824 |
| Rad-model | 2.72 (1.93, 3.94) | 2.82 (2.05, 3.83) | 0.783 |

Comparison of demographic, clinical, laboratory, and baseline Agatston score between the training and validation cohorts. Data are presented as median (interquartile range), or number (percentage), as appropriate. P-values were

derived using Mann-Whitney U tests, or Chi-square tests.

Abbreviations: SBP, Systolic Blood Pressure; DBP, Diastolic Blood Pressure; BMI, body mass index; AST, Aspartate Aminotransferase; ALT, Alanine Aminotransferase; eGFR, Estimated Glomerular Filtration Rate; Glu, Glucose; TG, Triglyceride; TC, Total Cholesterol; HDL, high-density lipoprotein; LDL-C, low-density lipoprotein cholesterol; hs-CRP, high-sensitivity C-reactive protein.

Table S2. Complete List of Extracted Radiomics Features and Their Reproducibility

| | **Original** | **Wavelets transformations (n=8)** | **log-sigma** | **All** |
|---|---|---|---|---|
| **First order** | 18 | 126 | 90 | 252 |
| **Shape-related** | 14 | - | - | 14 |
| **GLCM** | 24 | 168 | 120 | 336 |
| **GLDM** | 14 | 198 | 70 | 196 |
| **GLRLM** | 16 | 112 | 80 | 224 |
| **GLSZM** | 16 | 112 | 80 | 224 |
| **Total** | 102 | 704 | 440 | 1246 |

GLCM: gray level co-occurrence matrix; GLDM: gray level dependence matrix; GLRLM: gray level run length matrix; GLSZM: gray level size zone matrix; NGTDM: neighbouring gray tone dependence matrix; PVAT: perivascular adipose tissue.

Catalog of the 1246 radiomics features extracted from perivascular adipose tissue regions using PyRadiomics.

Table S3. Baseline Characteristics of the Plaque Progression Subgroup

| Characteristic | Progression cohort (n=60) | Remaining cohort (n=482) | p-value |
|---|---|---|---|
| Age (years) | 62.00 (51.00, 70.00) | 61.00 (54.00, 66.00) | 0.452 |
| Sex (male) | 39 (65.0) | 350 (72.6) | 0.221 |
| BMI (kg/m2) | 25.50 (23.70, 27.70) | 25.75 (23.85, 27.90) | 0.634 |
| Hypertension (%) | 40 (66.7) | 313 (64.9) | 0.785 |
| Hyperlipidemia (%) | 48 (80.0) | 385 (79.9) | 0.985 |
| Diabetes (%) | 21 (35.0) | 175 (36.3) | 0.841 |
| eGFR (mL/min/1.73m) | 96.51 (87.22, 103.57) | 93.28 (83.16, 101.26) | 0.412 |
| Glu (mmol/L) | 6.08 (5.51, 7.14) | 5.98 (5.10, 7.68) | 0.512 |
| TG (mmol/L) | 1.36 (0.95, 2.02) | 1.45 (1.08, 2.06) | 0.615 |
| TC (mmol/L) | 4.23 (3.56, 5.12) | 4.23 (3.42, 5.03) | 0.887 |
| HDL-C (mmol/L) | 1.14 (0.98, 1.34) | 1.04 (0.89, 1.22) | 0.345 |
| LDL-C (mmol/L) | 2.42 (1.85, 3.13) | 2.25 (1.78, 3.05) | 0.388 |
| hsCRP (mg/L) | 1.47 (0.62, 3.63) | 1.23 (0.63, 2.80) | 0.415 |
| Agatston score | 386.42 (101.19, 571.11) | 346.38 (75.29, 890.26) | 0.511 |
| Rad-model | 4.28 (3.18, 6.07) | 4.15 (3.05, 5.95) | 0.756 |

Comparison of demographic, clinical, laboratory, and baseline Agatston score between the progression cohort and remaining cohort. Data are presented as median (interquartile range), or number (percentage), as appropriate. p-values were derived using Mann–Whitney U tests, or Chi-square tests. Abbreviations: BMI, body mass index; eGFR, Estimated Glomerular Filtration Rate; Glu, Glucose; TG, Triglyceride; TC, Total Cholesterol; HDL, high-density lipoprotein; LDL-C, low-density lipoprotein cholesterol; hs-CRP, high-sensitivity C-reactive protein.

## Supplement Figures

Figure S1. Feature Stability analysis

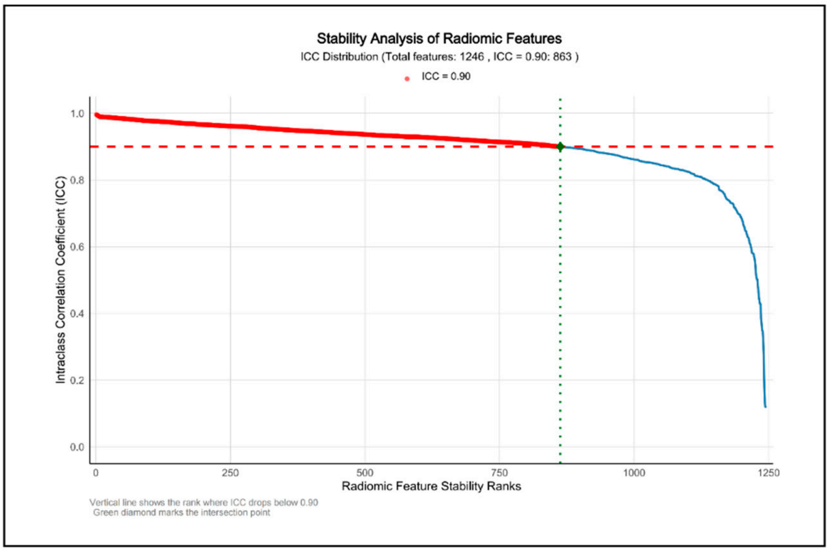


Distribution of intraclass correlation coefficients (ICCs) for all 1246 extracted features; the dashed line indicates the ICC > 0.9 threshold for good reproducibility

Figure S2. Feature Selection Process

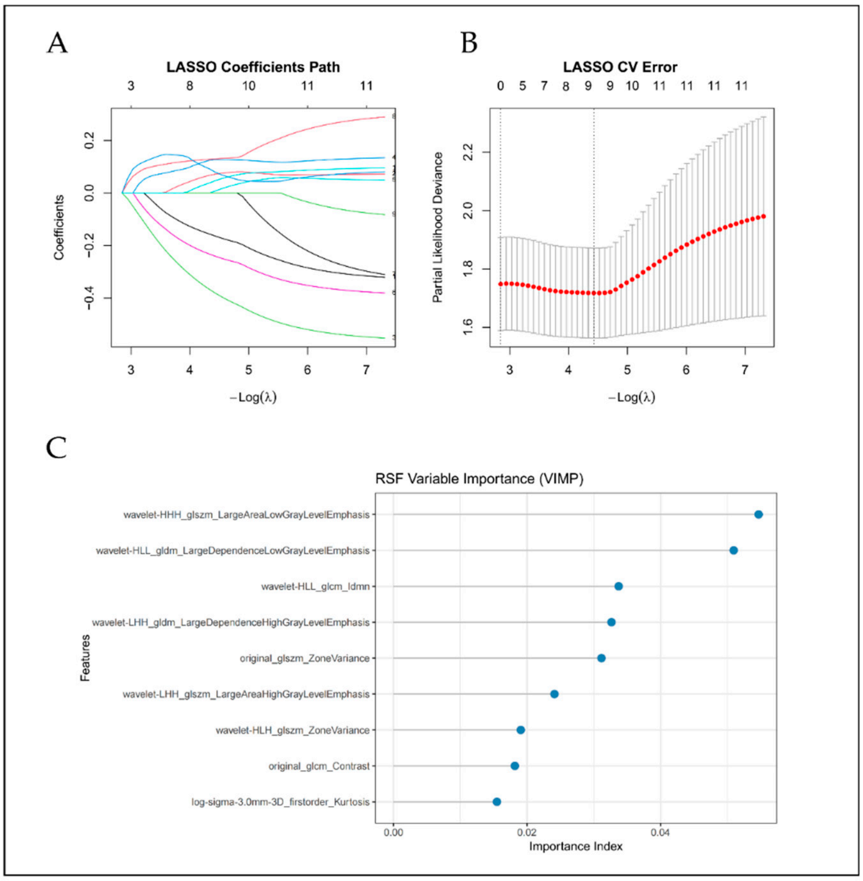


(A) LASSO coefficient profiles. (B) Cross-validation curve for selection of the optimal lambda (λ) parameter (right).

Vertical dashed lines indicate λ.min (minimum error) and λ.1se (most regularized model within 1 SE of minimum).

(C) Variable importance ranking from the random survival forest (RSF) analysis in the training set.
